# Anthocyanin Biosynthesis and DNA Methylation Dynamics in Sweet Orange Fruit [*Citrus sinensis* L. (Osbeck)] under Cold Stress

**DOI:** 10.1021/acs.jafc.0c02360

**Published:** 2020-06-10

**Authors:** A. Sicilia, E. Scialò, I. Puglisi, A. R. Lo Piero

**Affiliations:** Department of Agriculture, Food and Environment (Di3A), University of Catania, Via Santa Sofia 98, 95123 Catania, Italy

**Keywords:** *Citrus sinensis*, sweet orange, anthocyanin, pigment variegation, DNA methylation, gene expression, cold stress

## Abstract

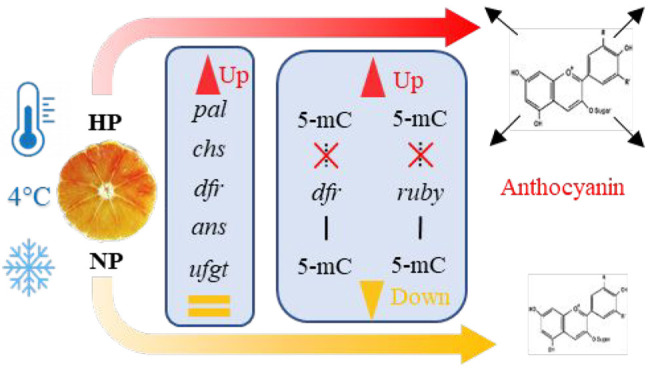

The blood red color of pigmented orange fruit varieties [*Citrus sinensis* L. (Osbeck)] is due to the presence of anthocyanin pigments that largely contribute to determine the high organoleptic qualities and the nutritional properties of the fruits. The content of pigments in sweet orange depends primarily on genetic factors and on environmental conditions. In particular, it has been extensively shown that cold temperature induces an increase of anthocyanin content that is achieved by the induction of the related gene expression. The purpose of our work is to understand the mechanism underlying the color variegation occurring inside the blood oranges during the cold induction of anthocyanin biosynthesis, despite the fact that the entire fruit is genotypically programmed to produce pigments. Therefore, the amount of anthocyanin and the expression of both structural and regulatory genes have been monitored in either high-pigmented (HP) or not/low pigmented (NP) segments of the same fruit during the storage at 4 °C for a total experimental period of 25 days. Our results clearly indicate that the anthocyanin content is directly correlated with the levels of gene transcription, with higher pigmented areas showing higher enhancement of gene expression. Furthermore, we analyzed the reshaping of the DNA methylation status at the promoter regions of genes related to anthocyanin biosynthetic pathway, such as *DFR* and *Ruby*. Our results unequivocally demonstrate that in the promoter regions of both *DFR* and *Ruby*, the amount of cytosine methylation strongly decreases along the cold storage in the HP areas, whereas it increases in the NP areas of the same fruit, probably causing a partial block of the gene transcription. Finally, by measuring the changes in the expression levels of the *Citrus* DNA demethylases, we found that DML1 might play a crucial role in determining the observed demethylation of *DFR* and *Ruby* promoters, with its expression induced by cold in the HP areas of the fruits. This is the first report in which different levels of gene expression implicated in anthocyanin production in blood orange fruit is correlated with an epigenetic control mechanism such as promoter methylation.

## Introduction

Anthocyanins are water-soluble pigments belonging to the flavonoid compound family concerned in numerous aspects of plant development and defense. Several varieties of sweet orange [(*Citrus sinensis*) L. Osbeck], which include Tarocco, Moro, and Sanguinello are able to synthesize anthocyanins which confer to the fruit the characteristic bloody color and make them easily distinguishable from the nonpigmented, blond orange varieties.^1^ Anthocyanins are synthesized via the flavonoid pathway, which is a ubiquitous and well-described plant secondary metabolism pathway.^2^ Most genes encoding the enzymes that biosynthesize the pigment molecules have been cloned and characterized in various species,^3^ the *Citrus* species included.^1,4−7^ Phenylalanine is a direct precursor for the synthesis of anthocyanidins and its conversion to the pigment core structure requires a series of reactions. The first of them is represented by the transformation of phenylalanine to *trans*-cinnamic acid catalyzed by the phenylalanine ammonia lyase (PAL). In the following steps, 4-hydroxylation of cinnamic acid by cinnamate 4-hydroxylase (C4H) generates *p*-coumaric acid that is converted by the 4-coumarate: CoA ligase (4CL) to the respective CoA ester. The first specific enzyme of the anthocyanin biosynthetic pathway is chalcone synthase (CHS) which condenses three malonyl-CoA and one *p*-coumaroyl-CoA molecules to produce the naringenin chalcone. The naringenin chalcone is isomerized by chalcone isomerase (CHI) to the flavanone naringenin, which is subsequently converted to dihydrokaempferol by flavanone 3′-hydroxylase (F3H). The dihydroflavonols, dihydroquercetin, and dihydromyricetin, are synthesized from dihydrokaempferol by sequential hydroxylations catalyzed by flavonoid 3′-hydroxylase (F3′H) and flavonoid 3′,5′-hydroxylase, respectively. Dihydroflavonol 4-reductase (DFR) can reduce the dihydroflavonols to their corresponding leucoanthocyanidins. Anthocyanidin synthase (ANS) converts the colorless leucoanthocyanidins into the colored anthocyanidins, that, once formed, are immediately modified by the UDP-glucose-flavonoid glucosyl transferase (UFGT) that catalyzes the addition of a glucose molecule in the 3-OH positions of anthocyanidins increasing their hydrophilicity and stability.^1^ A similar mechanism to the detoxification process was proposed for anthocyanin transfer into the vacuole, this localization being necessary to prevent oxidation.^8,9^ According to that, anthocyanins need to be conjugated to glutathione by a specific glutathione transferase (GST)^6^ that is often indicated as the real last enzyme involved in this metabolic pathway. Anthocyanin biosynthesis is regulated mainly at the transcriptional level by the MBW complex, composed by proteins of the Myb, basic helix–loop–helix (bHLH) and WD-repeat families of transcription factors.^3,10,11^ Specifically, pigmentation of blood oranges originates from a retrotransposon insertion that allows the expression of *Ruby* encoding the MYB-type transcription factor of the MBW complex that activates anthocyanin production in *C. sinensis*.^11^ Accordingly, the majority of differences in anthocyanin levels in *Citrus* are related with the alteration of *Ruby* expression caused by point mutations, or by *indels* resulting from transposable elements.^12^ Recently, a group of mutants have been identified in which anthocyanin pigmentation does not occur despite the presence of wild-type *Ruby* alleles. This subset of anthocyaninless variants is characterized by the absence of proanthocyanidins in the seeds, and by fruit juice almost completely lacking in acidity,^13^ together defining the “acidless” phenotype. A bHLH gene named *Noemi* has been found to have a crucial role in the control of flavonoid biosynthesis as it interacts with *Ruby* to determine both anthocyanin production in citrus leaves and bud and the regulation of fruit acidity.^13^ However, the role of *Noemi* in pigmented sweet orange fruit has not yet been investigated either in normal or in stressful conditions. In the pigmented varieties, the anthocyanin content may also vary in response of different environmental conditions as such as light exposure, nutritional status, xenobiotic or hormone treatments, and temperature.^14−19^ The cold induction of anthocyanin biosynthesis has received greater interest than other inducing environmental challenges because the productivity of commercially important *Citrus* varieties is seriously laid down by low temperature that induces metabolic changes such as elevated electrolyte leakage, reduced photosynthetic capacity, and respiration rate.^20^ Most interestingly, it has been shown that the pigment content of freshly harvested fruits significantly increases throughout cold storage due to ongoing biosynthesis of anthocyanins.^14,16,17^ This enhancement is accomplished by both the up regulation of the genes involved in the anthocyanin biosynthesis^14,16,17^ and the increase of encoded proteins,^19^ as well as by the induction of the regulatory *Ruby* gene transcription.^11^ Further transcriptome study showed that cold stress induces transcriptome adjustments absolutely orientated toward the enhancement of the flavonoid biosynthesis pathway in blood oranges, including those genes belonging to upstream metabolic pathways, such as the shikimate pathway that yields phenylalanine.^16^ Interestingly, the red color that is achieved in fruit flesh during both ripening and in response to cold is not uniformly distributed in the fruit flesh. On the contrary, pigment accumulation is concentrated in particular areas of the entire fruit in which it is then possible to observe highly pigmented and low/not pigmented segments, despite the fact that they are programmed to synthesize anthocyanins by their genotype. DNA methylation, in combination with histone modifications and non-histone proteins, defines chromatin structure and accessibility. DNA methylation contributes to regulate gene expression, transposon silencing, chromosome interactions, and trait inheritance. Many early studies of abiotic stress showed stress-induced DNA methylation and/or demethylation patterns either genome wide or at specific loci. In some cases, these changes in DNA methylation may be associated with transcriptional regulation of genes involved in plant stress responses^21−24^ suggesting that DNA methylation is important in mediating plant interaction with the environmental signals. In this work, we evaluated the effect of cold temperature storage upon anthocyanin accumulation and the related gene expression both in high-pigmented (HP) and low/no pigmented (NP) areas of the same fruit, separately. Similarly, Methylation Sensitive Amplification Polymorphism (MSAP) analysis was used to determine the methylation status of the DFR and *Ruby* promoters during cold storage in both NP and HP areas to correlate the DNA methylation levels with gene expression. In this way, we tried an easy way to set to zero the genetic differences (same fruit) in order to highlight the variation in epigenetic marks that can be responsible of different gene expression levels. Moreover, the expression of DNA demethylases involved in the dynamics of methylation rearrangements was also measured. Finally, the role of the BHLH *Noemi* gene in the variegation of pigmentation during cold stress was considered and discussed.

## Materials and Methods

### Plant Material and Cold Storage Conditions

Pigmented oranges (Tarocco Tapi) [*C. sinensis* (L.) Osbeck] were harvested in January 2019 from approximately 15 year old trees grown at the experimental agricultural field of University of Catania located in Primosole, in the territory of Catania (Italy). Freshly harvested oranges were washed with distilled water, gently dried with paper towels, and then left to dry at room temperature overnight. Subsequently, orange fruits were placed in a box (60 fruits) stored in a ventilated cold room at 4 °C and 90–95% relative humidity (RH). Samplings of the cold stored fruits were carried out every 5 days for a total storage period of 25 days. During each sampling, 9 fruits were randomly collected and divided into three subgroups of three fruits each. After fruit peeling, orange wedges have been cut. The highly pigmented areas of the wegdes were separated from the low/non- pigmented areas and those belonging to different fruits of the same subgroup were pooled to constitute three independent mean samples, and indicated as high-pigmented (HP) and low/no pigmented (NP). The orange flesh was then immediately frozen with liquid nitrogen and stored at −80 °C until used (both RNA and DNA extraction and anthocyanin determination).

### Extraction of Total RNA and cDNA Synthesis

The total RNA from orange fruit flesh (highly pigmented, HP, and low/no pigmented, NP areas) was extracted using the RNAesy kit (Qiagen). Reverse transcription was achieved using 2 μg of total RNA as the starting material using the SuperScriptTM ViloTM cDNA synthesis kit by ThermoFisher Scientific, according to the manufacturer’s instructions.

### Measurement of Gene Expression by Real-Time Quantitative RT-PCR

Real-time qRT-PCR was performed with PowerUp SYBR Green Master mix by ThermoFisher Scientific and carried out in the Bio-Rad iQ5 Thermal Cycler detection system. Primers, whose sequences are shown in Table 1, were designed using the Eurofins genomics “*PCR Primer Design Tool*” and obtained therein. The relative quantitation of gene expression of the structural genes involved in anthocyanin biosynthesis (*pal*, *chs*, *dfr*, *ans*, and *ufgt*), in the regulation of anthocyanin biosynthesis (*Ruby* and *Noemi*), in DNA demethylation (*dme*, *dml1*, *dml3*, and *dml4*), was performed in triplicate and the fold change measurements calculated with the 2^–ΔΔCT^ method as described in Lo Piero et al.^14^ The elongation factor EF-1α housekeeping gene was used as an endogenous reference. The ΔΔ*C*_T_ was calculated by subtracting the baseline’s Δ*C*_T_ to the sample’s Δ*C*_T_ where the baseline represents the expression level in the low/not pigmented (NP) areas. Negative controls without reverse transcriptase were routinely included.

**Table 1:** Primer Sequences for Analysis of Gene Expression and MSAP

primer	sequence
*Cs*_CHS_For	5′-TCTATGGACGGGCATCTTC-3′
*Cs*_CHS_Rev	5′-TGCCTCGGTTAGGCTTTTC-3′
*Cs*_DFR_For	5′-GCTGTTCGTGCTACTGTTC-3′
*Cs*_DFR_Rev	5′-GGCTAAATCGGCTTTCCATA-3′
*Cs*_ANS_For	5′- GGTGACTGCTAAATGTGTT-3′
*Cs*_ANS_Rev	5′CAAGTCCCCTGTGAAGAATA-3′
*Cs*_UFGT_For	5′- TCTTCAGCACTCCGCAATC-3′
*Cs*_UFGT_Rev	5′-TCCATCGGATACGTCGTAAG-3′
*Cs*_*Ruby*_For	5′-ACAATCCACCCCGTCTGATC-3′
*Cs*_*Ruby*_Rev	5′-CTGGCCTGCTTCAATGACTC-3′
*Cs*_DME_For	5′-CAGAAACCGCCCAAACGAAG-3′
*Cs*_DME_Rev	5′-GCATCGGTTGTCTCCCTGAT-3′
*Cs*_DML1_For	5′-GCCGCAGAATCCACTAACCT-3′
*Cs*_DML1_Rev	5′-CTTTACACAGCTGCCCGGTA-3′
Cs_DML3_For	5′-CGGCGAAAAAGCAACTCCAA-3′
*Cs*_DML3_Rev	5′-CTATGGTTCTGCCAGCGACA-3′
*Cs*_DML4_For	5′-GAAACCAGGCAAGACCCGTA-3′
*Cs*_DML4_Rev	5′-TTCATATCCAACGGCACGCT-3′
*Cs*_*Ruby*_ Pro2_For	5′-CGATGGAGTTTGGGCTTGAG-3′
*Cs*_*Ruby_* Pro2_Rev	5′CCAGTCCAAGTTAACAATTCCCA-3′
*Cs*_DFR_pro_For	5′-ACCCAAAAGTAGGCCCAAGT-3′
*Cs*_DFR_pro_Rev	5′-GTTGCCGGGCTTGTTTATGT-3′

### Total Anthocyanins Content

Anthocyanin determination was performed on 1 g of orange flesh (separately on HP and NP segments) by pH differential spectrophotometry according to the method described in Lo Cicero et al.^25^

### Methylation Sensitive Digestion

DNA extraction was performed upon samples belonging to the HP and NP areas of the same fruit average sample using the Invitrogen Plant DNAzol reagent kit. The quality and integrity of extracted DNA was evaluated by spectrophotometric analysis at NanoDrop (ThermoFisher Scientific, Waltham, MA, U.S.A.) and trough gel electrophoresis, respectively. DNA digestion was carried out using the methylation-sensitive endonuclease McrBC (New England Biolab Inc., Ipswich, U.S.A.) which cleaves DNA containing methylcytosine and 5-hydroxymethylcytosine on one or both strands. Sites on the DNA recognized by McrBC consist of two half-sites of the form (G/A)mC. These half-sites can be separated by up to 3 kb, but the optimal separation is 55–103 base pairs.^26^ The digestion was carried out according to the following conditions: NEBuffer 2 (1×), BSA (200 μg/mL), GTP (3 mM), McrBC (15 U), 500 ng DNA, ddH_2_O up to 25 μL, at 37 °C for 8 h followed by enzyme inactivation at 65 °C for 20 min. McrBC requires GTP for cleavage.^27^ For each sample, a “test reaction” in which GTP is included in the digestion mix and a “reference reaction” in which GTP is excluded were prepared. Successively, real-time PCR was performed using the PowerUpTM SYBR Green Master Mix according to the manufacturer’s instructions: 40 ng of digested DNA was mixed with master mix 1×, 400 nM of each primer in a total volume of 20 μL. Primers sequences are listed in Table 1 and indicated by wording “*pro*”. A “test reaction” and a “reference reaction” were prepared by adding DNA from “test reaction” and “reference reaction” of the previous digestion step, respectively. Each sample was screened in triple technical replicates. The mean *C*_T_ values obtained were used to calculate Δ*C*_T_ as follows: Δ*C*_T_ = [*C*_T_(test) – *C*_T_(reference)] and the methylation percentage was calculated as methylation% = 100 – (100 × 2^–Δ*C*_T_^).

### Statistical Analysis

Data were analyzed by one-way ANOVA (*p* < 0.05) followed by Tukey’s test for multiple comparison procedures using the statistical software package Statistica v. 13.0 (Dell Inc., Round Rock, TX, U.S.A.).

## Results and Discussion

**Figure 1:**
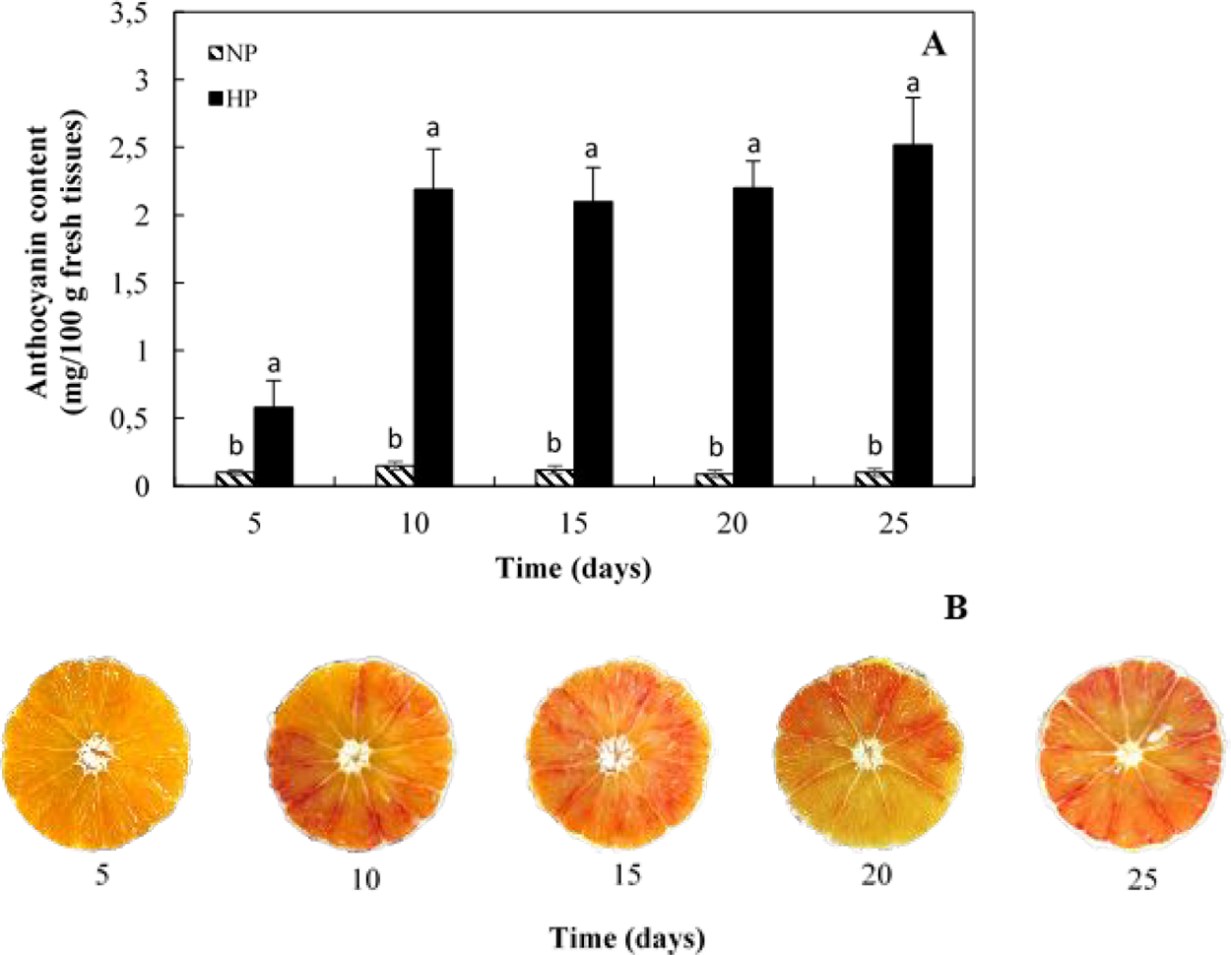
(A) Anthocyanin content in pigmented (HP) and low/not pigmented (NP) areas of cold stored fruit flesh; (B) picture of sweet orange cold stored fruits. Samples have been prepared as described in the “Materials and Methods” section and assayed for anthocyanin content. Each point represents the mean value of three replications ± SD. Each replication was composed of three fruits. Significantly different values, within each sampling time, are indicated by different letters (*p* < 0.05).

**Figure 2:**
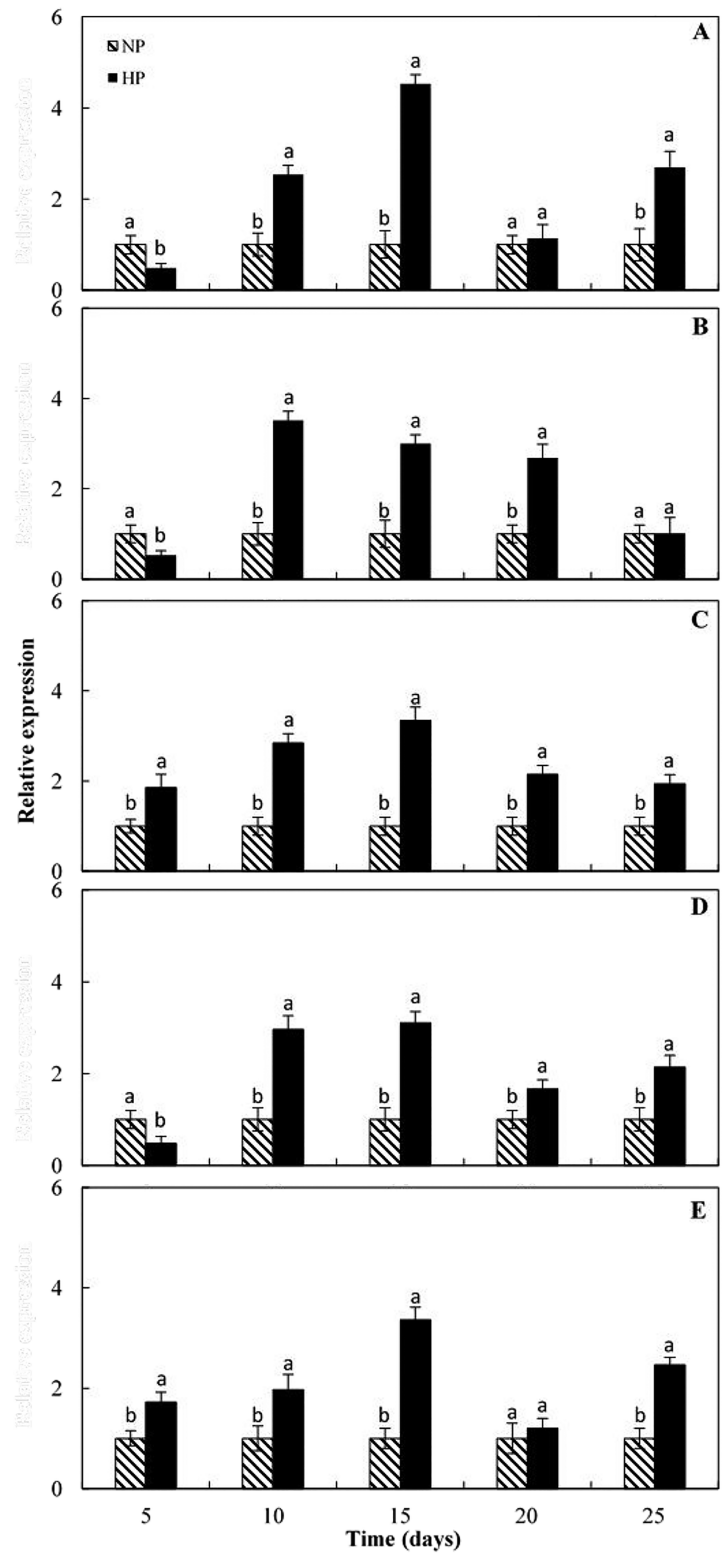
Evaluation of gene expression in high pigmented (HP) and low/not pigmented areas (NP) of fruit flesh during cold storage (A) *pal*, (B)*chs*, (C)*dfr*, (D)*ans*, and (E)*ufgt*. The relative quantitation of gene expression between samples was calculated by real time RT-PCR using the comparative threshold (*C*_T_) method, as described in the Materials and Methods. The ΔΔ*C*_T_ was calculated by subtracting the baseline’s Δ*C*_T_ to the sample’s Δ*C*_T_ where the baseline represents the expression level in the low/not pigmented areas. Each point represents the mean value of three replications ± SD. Each replication was composed of three fruits. Significantly different values, within each sampling time, are indicated by different letters (*p* < 0.05).

**Figure 3:**
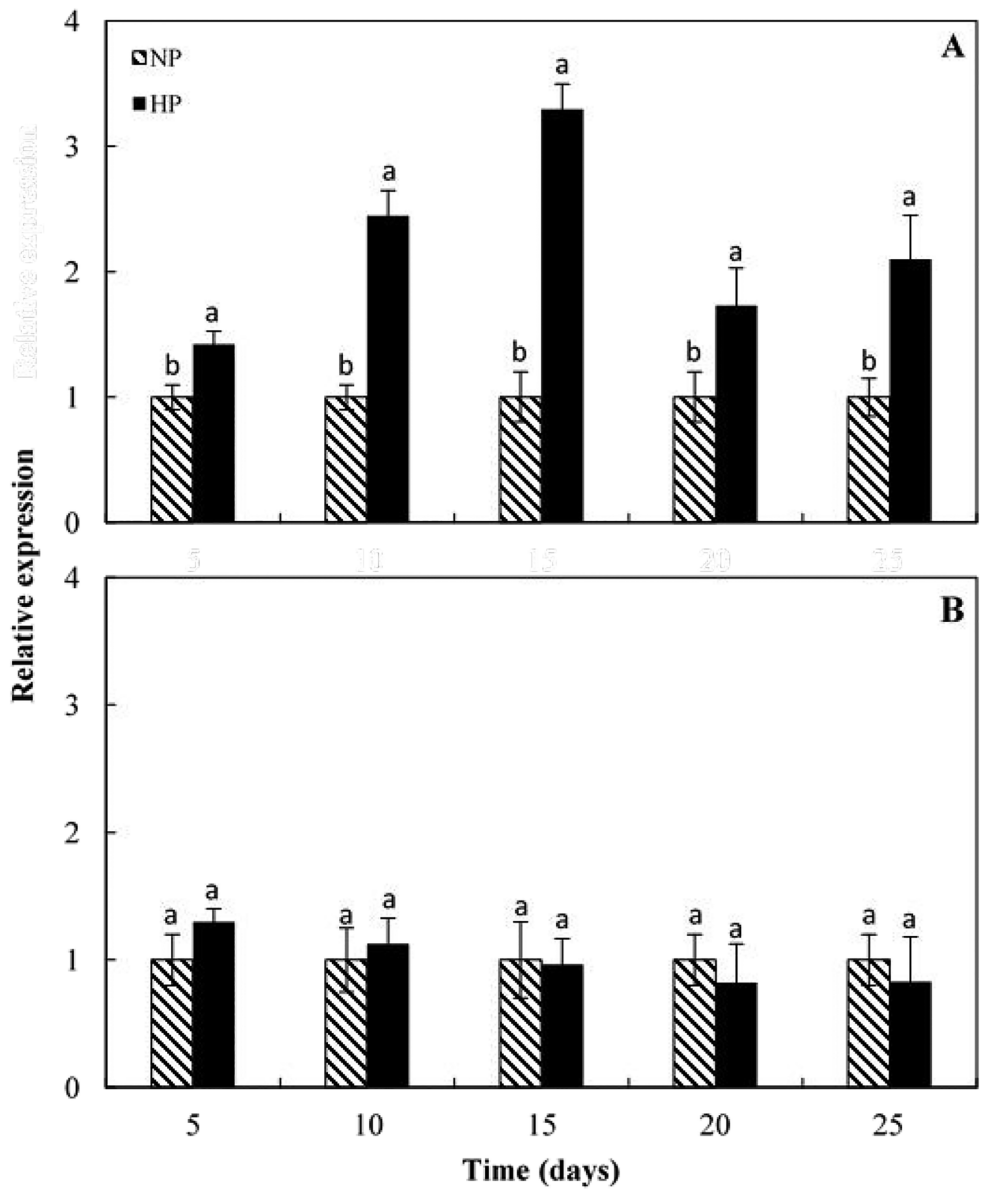
Expression analysis of regulatory genes (A)*Ruby*, (B) *Noemi*, in high pigmented (HP) and low/not pigmented (NP) areas of fruit flesh during cold storage by real time RT-PCR. For details, refer to “Materials and Methods”. Each point represents the mean value of three replications ± SD. Each replication was composed of three fruits. Significantly different values, within each sampling time, are indicated by different letters (*p* < 0.05).

**Figure 4:**
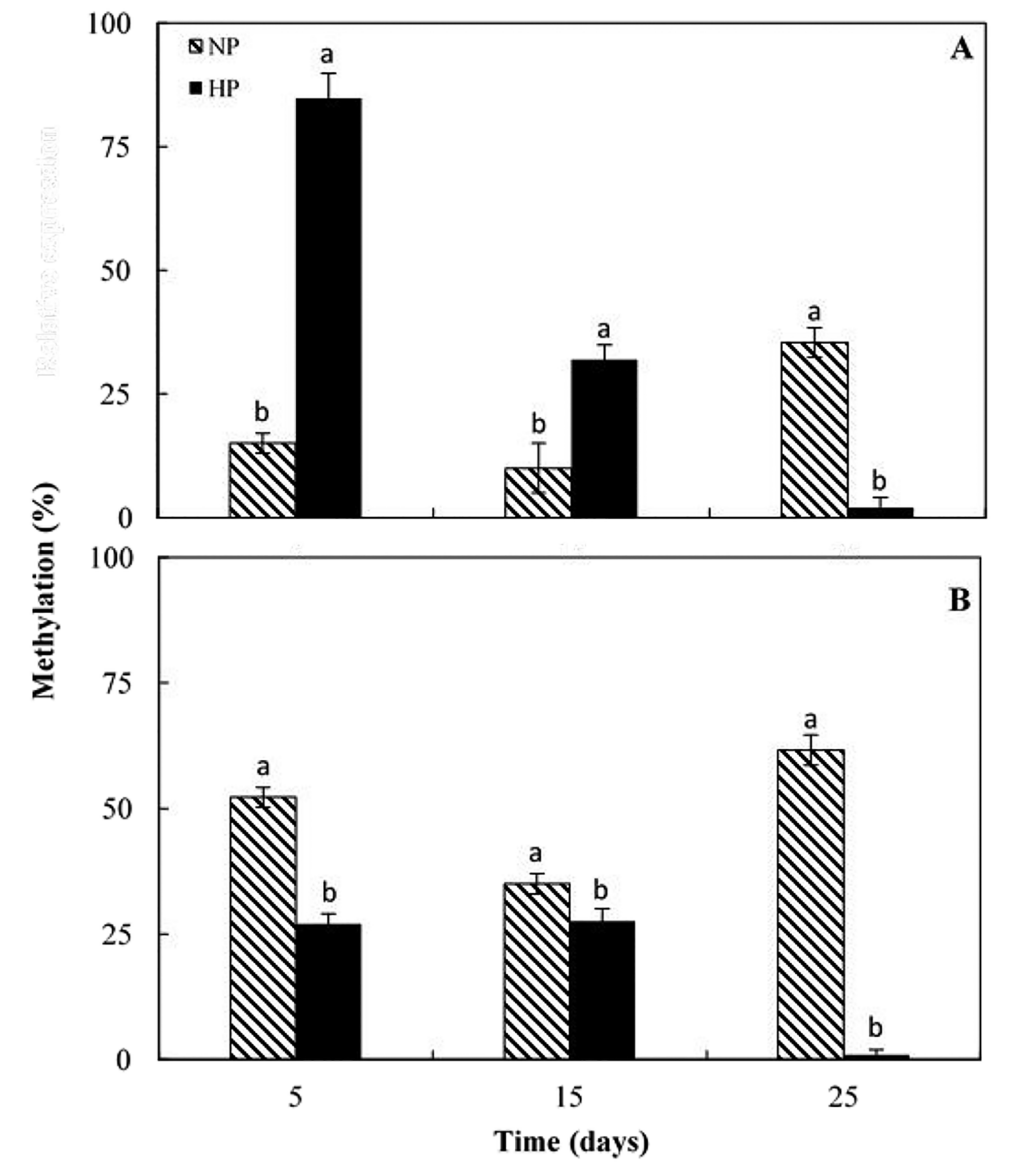
Analysis of the methylation status of both *Ruby* (A) and *DFR* (B) promoter regions. The levels of (G/A)mC was measured in both pigmented (HP) and not/low pigmented (NP) areas of fruit flesh at 5, 15, and 25 days storage by the application of the MSAP technique. For details, refer to “Materials and Methods”. Each point represents the mean value of three replications ± SD. Each replication was composed of three fruits. Significantly different values, within each sampling time, are indicated by different letters (*p* < 0.05).

**Figure 5:**
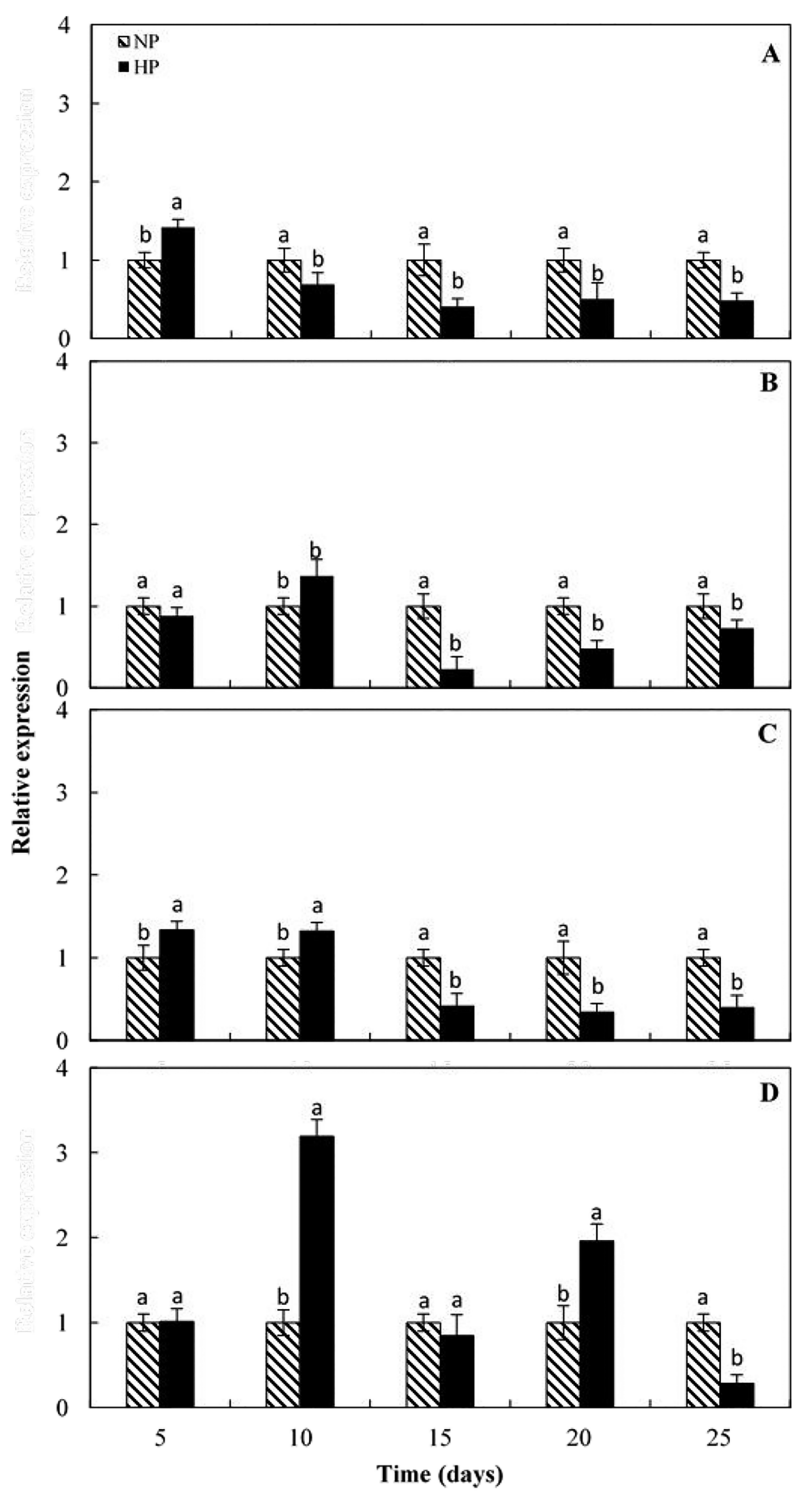
Expression pattern of demethylase encoding genes (A)*dme*, (B)*dml3*, (C)*dml4*, and (D)*dml1*in pigmented (HP) and low/not pigmented (NP) areas of fruit flesh during cold storage. For details, refer to “Materials and Methods” Each point represents the mean value of three replications ± SD. Each replication was composed of three fruits. Significantly different values, within each sampling time, are indicated by different letters (*p* < 0.05).

The analysis of the pigmentation of the blood orange varieties has been subject of many researches and has clarified the pathway leading to anthocyanin biosynthesis and its regulation.^1^ Presently, it is well-known that anthocyanin pigmentation is exclusively achieved in the blood orange varieties because of a transposon insertion at the *Ruby* regulatory gene promoter that allows *Ruby* expression as well as the constitution of the MBW complex needed for the activation of the biosynthetic metabolic pathway.^11^ The effect of low temperature upon the anthocyanin biosynthesis has also been studied in depth, ascertaining that pigment levels increase in response to cold storage due to the induction of structural genes involved in their biosynthesis,^1,4−6^ as well as to the enhancement of *Ruby* expression.^11^ However, the reason why different levels of pigment content are achieved inside the same orange fruit has been unexplored, and it could lead to novel knowledge in the regulation of pigment production. In this work, the amount of anthocyanin was measured in two separated segments of the same orange fruit characterized by a different level of pigmentation, and indicated as highly pigmented (HP) and low/no pigmented (NP) areas. The effect of cold storage upon the anthocyanin content of both HP and NP segments of orange fruits is reported in Figure 1. The amount of anthocyanins of NP samples was not affected by cold during the entire experimental period. On the contrary, the anthocyanin content of the HP areas sharply increased starting from the second sampling date throughout the remaining storage period (Figure 1). Pigment levels, in fact, rose from an initial value of 0.6 mg/100 g (5 days of storage) to 2.46 mg/100 g (after 25 days storage). The amount of anthocyanin was also measured at the third (15 days of storage) and at fifth (25 days of storage) samplings upon flesh samples left at room temperature (25 °C). In those samples, the anthocyanin level did not increase, thus indicating that the observed increase of pigment levels in HP areas of cold stored fruit is specifically induced by low temperature (data not shown). The expression profile of *pal*, *chs*, *dfr*, *ans*, and *ufgt* was investigated in the above-mentioned HP and NP segments of orange fruits exposed to cold using the real time RT-PCR. The results are illustrated in Figure 2 reporting the relative transcript levels of considered genes standardized to the constitutive elongation factor EF-1α gene expression level and normalized to NP samples 2^–ΔΔ*C*_T_^ (see the Materials and Methods for details). As shown in Figure 2A, the expression of *pal* increased from the first sampling date on (5 days storage) reaching a peak after 15 days of cold storage in the HP areas at which the expression level was more than 4 times higher with respect to the NP areas. Similarly, the transcripts of *chs*, *dfr*, *ans*, and *ufgt* enhanced up at the second sampling reaching at the third sampling (after 15 days of storage) expression levels ranging between 3 and 4 times higher than the NP samples (Figure 2B–E). Therefore, the different pigmentation, revealed by visual inspection in different areas of the same cold stored fruit, was assessed by direct anthocyanin measurement (Figure 1) and it is brought by increased expression levels of the structural genes involved in pigment biosynthesis (Figure 2). Figure 3 reports the expression pattern of the *Ruby* and *Noemi* regulatory genes. In particular, Figure 3A highlights that *Ruby* expression increases in the HP areas of the cold stored fruits reaching a peak after 15 days of storage at which it shows more than three times higher values of the NP areas, in line with the expression pattern of the structural genes. Hence, the metabolic pathway of anthocyanin biosynthesis involving both structural and *Ruby* regulatory genes is cold- induced in the HP pigmented areas. As concerns the expression pattern of *Noemi* during cold storage, Figure 3B clearly shows that no differences in gene expression can be registered between HP and NP areas of the same fruit during the entire experimental period. This finding suggests that although *Noemi* is expressed in the sweet orange flesh, it seems not implicated in the cold induced increase of anthocyanin content in the fruit HP areas. Consequently, our results contribute to define the picture on the *Noemi* function in *Citrus* genus which it is strictly correlated to both anthocyanin production in leaves and buds, and to high acidity in the fruit,^13^ but likely not in the control of pigmentation in fruit flesh. In this latter organ the activity of *Noemi* seems to be released by the role of *Ruby* in determine pigmentation, at least under cold stress. It has been shown that epigenetic marks such as DNA cytosine methylation can control the expression of several traits especially under stressful conditions, often overlapping their genetic control.^21^ Many studies have explored how DNA methylation regulates fruit development. The analysis of the methylome of tomato fruit during development at single base resolution revealed an epigenetic trigger of tomato fruit ripening represented by a gradual decrease of DNA methylation.^28^ However, it seems that there is a causal relationship between fruit ripening and DNA methylation. For instance, the single-base resolution DNA methylome of sweet orange fruits revealed that ripe oranges gain a global increase in DNA methylation during fruit ripening indicating that the DNA hypermethylation is critical for the proper ripening of oranges as well as of Satsuma mandarin fruits.^29,30^ Gene-associated DNA methylation in plants can occur in the promoter region or within the transcribed gene body. Genes with methylated promoters have a higher degree of tissue-specific expression than other genes.^31^ Additionally, promoter DNA methylation typically inhibits gene transcription, even though it can also promote gene expression, as it happens in the case of several ripening-repressed genes, such as those involved in photosynthesis, in mutant tomato fruits lacking of demethylase activity (SlDML2).^32^ Promoter DNA methylation directly represses gene transcription by affecting the binding of transcription activators to DNA or promoting the binding of transcription repressors or, indirectly, by inducing repressive histone modifications.^33,34^ In Figure 4, the variation of methylation status of *Ruby* and *DFR* promoters during cold stress is shown in HP and NP fruit segments by MSAP, as described in the Materials and Methods section. Besides *Ruby*, we chose DFR as it represents an important regulatory point in the anthocyanin biosynthetic pathway, also controlling the flux into biosynthetic pathway branches leading to distinct anthocyanin profiles.^5^ The analysis of methylation status of *Ruby* promoter highlighted that the percentage of DNA methylation increased in the NP segments from 15% to 35% during cold stress, whereas a dramatic reduction of the *Ruby* promoter methylation is observed in the HP areas during the experimental period (Figure 4A). Although the methylation status of the *Ruby* promoter is higher after 5 days of cold storage in the HP portion of the fruit, it shows a gradual and sharp reduction indicating that cold stress interferes with the programmed methylation dynamics inside the fruit by inducing demethylation and causing the activation of the anthocyanin biosynthesis. Similarly, hypomethylation of the *Md*MYB1 promoter, regulating anthocyanin production in apples, was correlated with the formation of red pigmentation in fruit skins during a bagging experiment aimed to enhance the red pigmentation in apple skin.^35^ Likewise, the *dfr* promoter was methylated (about 55%, after 25 days of storage) in the NP areas, whereas a decrease of methylation status is registered in the HP segments reaching the 1% value at the end of the experimental period (Figure 4B). Therefore, cold stress induces deep and different rearrangements of the methylation at *Ruby* and *DFR* promoter regions that result strongly demethylated in the HP areas compared to the NP areas, at the end of the cold exposure. Consequently, it is likely that DNA demethylation at *Ruby* and *DFR* promoters might have a crucial role in “*opening*” of the anthocyanin biosynthesis pathway induced by cold in the HP segments of orange fruits. On the contrary, the maintenance of high levels of DNA methylation might represent a partial block of gene transcription in the NP segments. DNA demethylation in *Arabidopsis* is catalyzed by 5′-methylcytosine DNA glycosylase/lyase enzymes, including REPRESSOR OF SILENCING 1 (ROS1), DEMETER (DME), DEMETER-LIKE 2 (DML2), and DML3.^36^ By searching for DNA demethylase genes in the orange genome, four *At*ROS1 orthologs have been identified indicating that distinct demethylases are involved in removing the DNA methylation mark in *Citrus* genus.^29^ They include orange1.1t01511 (*Cs*DME), Cs6g15500 (*Cs*DML1), Cs5g04950 (*Cs*DML4), and Cs3g07800 (*Cs*DML3). The expression of these four genes was monitored in both HP and NP areas of cold stressed samples. As shown in Figure 5A–C, the expression level of DME, DML4, and DML3 was repressed by cold in the HP segments compared to NP areas starting from the 15 days of storage. Conversely, the expression of DML1 was induced by cold in the HP areas in correspondence of the second (10 days) and fourth (20 days) samplings (Figure 5D). Therefore, the increase in the expression of DNA demethylase DML1 gene is highly consistent with the gradual decrease in DNA methylation levels of the HP segments during cold storage and suggests a main role of DML1 in remodeling the methylation status of those areas. The importance assumed by the DNA demethylases in the reshaping of the DNA methylation status has been also observed during Satsuma fruit ripening, in which the global increase in DNA methylation is likely due to decreasing in the expression of DNA demethylase genes.^30^ In conclusion, we assessed that cold stress actives the anthocyanin production in blood oranges, which are genotypically programmed to do so, not evenly, but determining differently pigmented segments inside the same fruit (Figure 1B). The cold induced enhancement of anthocyanin is caused by the ongoing of induction of regulatory (*Ruby*) and structural (*pal*, *chs*, *dfr*, *ans* ,and *ufgt*) gene expression.

Moreover, the analysis of methylation status of *Ruby* and *DFR* promoters indicated that the level of cytosine methylation sharply decreases in HP segments thus suggesting that DNA demethylation might represent a reversible epigenetic mark that is removed during cold stress in the HP areas. Finally, by the analysis of gene expression, it is likely that DML1 is the demethylase involved in the cold induced DNA demethylation occurred in the HP areas. Finally, the role of *Noemi* in fruit flesh under cold stress has been investigated indicating that it is not involved in cold induced fruit pigmentation. As far as we know, this is the first report regarding the role of the epigenetic control of fruit anthocyanin synthesis induced by cold that might be correlated with its dappled pigmentation.

## Abbreviations Used

PAL: phenylalanine ammonia-lyase
C4H: cinnamate 4-hydroxylase 4CL
4CL: 4-coumarate:CoA ligase
CHS: chalcone synthase
CHI: chalcone isomerase
F3H: flavanone 3′-hydroxylase
F3′H: flavonoid 3′-hydroxylase
F3′: 5′H, flavonoid 3′,5′-hydroxylase
DFR: dihydroflavonol-4-reductase
ANS: anthocyanidin synthase
UFGT: UDP-glucose flavonoid glucosyltransferase
EF: elongation factor
HP: high-pigmented
NP: low/no pigmented (NP)
